# The Correlation of the Neutrophil-to-Lymphocyte Ratio With Microvascular Complications in Patients With Diabetes Mellitus

**DOI:** 10.7759/cureus.44601

**Published:** 2023-09-03

**Authors:** Mayank Mahajan, Manoj Kumar Prasad, Chanchal Ashok, Rishi Tuhin Guria, Sujeet Marandi, Vidyapati, Sudhanshu Subrat, Anindya Chowdhury

**Affiliations:** 1 Medicine, Rajendra Institute of Medical Sciences, Ranchi, IND; 2 Internal Medicine, Rajendra Institute of Medical Sciences, Ranchi, IND; 3 Pathology, Rajendra Institute of Medical Sciences, Ranchi, IND

**Keywords:** neutrophil to lymphocyte ratio (nlr), diabetic neuropathy, diabetic retinopathy, diabetic nephropathy, neutrophil lymphocyte ratio, microvascular complications, diabetes mellitus

## Abstract

Background

High neutrophil-to-lymphocyte ratio (NLR) may be used as a reliable measure of vascular complications and an indicator of poor outcomes in cases of diabetes mellitus (DM).

Methods

A prospective analytical cross-sectional observational study was conducted at the Rajendra Institute of Medical Sciences (RIMS), Ranchi, Jharkhand, India. A total of 100 patients with DM who met the inclusion and exclusion criteria were included in the study. A pre-tested and semi-structured questionnaire was given to the patients. IBM SPSS software version 26 (IBM Corp., Armonk, NY, USA) and MedCalc trial version 20.114 (MedCalc Software Ltd., Ostend, Belgium) were used for data analysis. Logistic regression analysis was performed to determine the association of the NLR with microvascular complications.

Results

In our study, the male-to-female ratio was 1.78:1 (male: 64 (n)%, female: 36 (n)%). The mean age of our study population was 56.28 ± 13.24 years. Of 58 patients with microvascular complications, 34 had a high NLR, and 24 patients had a normal NLR. Of 42 patients without microvascular complications, only 14 had a high NLR, and the remaining 28 patients had a normal NLR (p = 0.012). Logistic regression was performed to analyze the association between the NLR and microvascular complications, which demonstrated a significant association (odds ratio (OR): 2.833, 95% confidence interval (CI): 1.238-6.481; p = 0.013).

Conclusions

Our study demonstrated the higher odds of having microvascular complications among diabetics with a high NLR compared with non-diabetics. Therefore, the NLR may be used as a measure of microvascular complications in the diabetic population.

## Introduction

The worldwide prevalence of type 2 diabetes is expected to increase to 7,079 per 100,000 individuals by 2030 [1]. According to the International Diabetes Federation, diabetes and its complications have resulted in approximately 6.7 million deaths in adults in 2021 [2]. Sustained hyperglycemia causes the linkage of sugars with lipids, proteins, and nucleic acids. Therefore, the accumulation of advanced glycation end products is increased in the blood vessels of the retina, glomerulus, and large blood vessel wall, resulting in microvascular and macrovascular complications.

The inflammatory process is involved in the pathogenesis of vascular complications. Chronic hyperglycemia causes oxidative stress in vessel walls through the oxidation of low-density lipoprotein, which results in changes in cell adhesion and increases the release of cytokines and growth factors. As the inflammatory response plays a central role in diabetic complications, there is a need to identify a marker as a reliable predictor, which may allow the early diagnosis of these complications.

The neutrophil-to-lymphocyte ratio (NLR) has emerged as an important indicator of inflammation and has been shown to have prognostic value for systemic inflammation in patients with chronic conditions [3]. The NLR is based on two markers: neutrophils (the initial line of defense) and lymphocytes (the regulatory component of inflammation) [4]. Because of its consistency and minimal influence from physiological and pathological factors, the NLR is superior to other leukocyte parameters [5]. The NLR is also a relatively cheaper and more accessible tool for evaluating inflammatory conditions. Hence, the NLR may be a prospective indicator of vascular complications and a predictor of poor outcomes in cases of diabetes mellitus (DM) [6]. A study by Shiny et al. stated that for patients with glucose intolerance, the NLR can be used as a supportive prognostic marker for microvascular and macrovascular complications [7]. Moursy et al. found that the NLR of patients with diabetic microvascular complications was higher than that of patients without microvascular complications and healthy subjects [8]. Kawamoto et al. investigated the association of the NLR with early kidney dysfunction and albuminuria in diabetics and concluded that the NLR could be important for evaluating diabetic patients with a higher degree of albuminuria [9]. However, there are limited studies on the role of NLR in managing diabetic patients. Thus, this study was conducted to determine the NLR of patients with diabetes and examine its association with microvascular complications in diabetes mellitus.

## Materials and methods

Study design

This was an analytical prospective cross-sectional observational single-center study. A total of 100 subjects with DM who met the inclusion and exclusion criteria were selected by consecutive sampling.

Setting

The study was conducted at the Rajendra Institute of Medical Sciences (RIMS) in Ranchi, Jharkhand, India, from July 2020 to August 2022.

Participants

The study population includes patients over 18 years old with a diagnosis of DM (new or old). We excluded pregnant patients, patients who were currently smoking and had a history of smoking, patients with a history of any immunological disease, and patients with a history of infectious disease in the week preceding enrollment. The included patients were from different wards at the RIMS. A pre-tested and semi-structured questionnaire was given to the patients.

Variables

Information on the duration of diabetes, any other associated diseases and symptoms suggestive of any complications, and treatment compliance was recorded. An NLR of >3.53 was considered high, and an NLR of <3.53 was considered normal [10]. Diabetes mellitus was diagnosed based on any of the following criteria: fasting plasma glucose ≥126 mg/dL (7.0 mmol/L) (the fasting state was defined as no caloric intake for at least eight hours), two hours post-prandial plasma glucose ≥200 mg/dL (11.1 mmol/L) during an oral glucose tolerance test (OGTT), glycosylated hemoglobin (HbA1c) ≥6.5%, or random plasma glucose ≥200 mg/dL (11.1 mmol/L) for patients with classic symptoms of hyperglycemia or hyperglycemic crisis [11]. Random spot urine tests were performed two or more times (three months apart), and a protein-to-creatinine ratio greater than three was used to determine the presence of diabetic nephropathy [12]. To check for sensation, a monofilament test (10 g) was performed, which was verified by nerve conduction velocity assessment [13]. A funduscopic examination was performed to detect diabetic retinopathy, which was grouped into proliferative diabetic retinopathy, non-proliferative diabetic retinopathy, and macular edema [14].

Data source/measurement

Patients included in the study were thoroughly examined for DM, diabetic nephropathy, neuropathy, and retinopathy, following the diagnostic criteria. A complete blood count (CBC) analysis was performed using an automated analyzer.

Bias

Double blinding was used to eliminate bias. Experts who were responsible for blood analysis, funduscopic examination, and monofilament testing were blinded. In addition, those who analyzed the data were blinded.

Study size

Based on the proportion of the population with diabetes in the study area and a confidence interval (CI) of 95%, our sample size was 100. The formula used was as follows: n = z2 p (1-p) / d2 = (1.96)2 × 0.073 × (1-0.073) / (0.05)2 = 100, where n is the sample size, z (1.96) is the statistic related to the level of confidence, p is the expected prevalence (7.3% as reported by Anjana et al. [15]), and d (0.05) is the precision.

Statistical method

The data were analyzed using IBM SPSS software version 26 (IBM Corp., Armonk, NY, USA) and MedCalc trial version 20.114 (MedCalc Software Ltd., Ostend, Belgium). Categorical variables are presented as the number and percentage (%), and continuous variables as the mean ± SD and median. The Kolmogorov-Smirnov test was used to test the normality of the data, and a non-parametric test was used if normality was rejected. Quantitative variables were evaluated by an independent t-test between the two groups. Qualitative variables were evaluated by the chi-square test. The receiver operating characteristic curve was used to determine the cut-off point of parameters for predicting various microvascular complications. To determine the odds ratio for microvascular complications, a logistic regression curve was used. A p-value of <0.05 was regarded as statistically significant.

Study approval and consent

The Institutional Ethical Committee of RIMS approved the study (memo no. 227, dated May 10, 21). Consent was obtained from each participant prior to enrollment in the study.

Ethical consideration

This study was conducted in accordance with the ethical principles for medical research involving human subjects outlined in the Declaration of Helsinki.

## Results

In our study, of the 100 subjects, 64 (n)% of them were male. The mean age of the study population was 56.3 ± 13.24 years. A total of 48 patients had a high NLR, whereas 52 patients had a normal NLR.

Of the 64 male patients, 29 patients had a high NLR. On the other hand, of the 36 female patients, 19 had a high NLR. In the chi-square test, the p-value was 0.475 (Table 1).

**Table 1:** Gender and NLR NLR: neutrophil-to-lymphocyte ratio; p-value: probability value

Gender	High NLR		Normal NLR		Combined		p-value
	Number of cases	%	Number of cases	%	Number of cases	%	
Male	29	45.31%	35	54.69%	64	100%	0.475
Female	19	52.78%	17	47.22%	36	100%	
Total	48	48.00%	52	52.00%	100	100%	

A total of 44 patients were diagnosed with DM within the last five years. Only five patients were diagnosed with DM for more than 15 years, and the longest period with diabetes was 20 years.

The mean period of diabetes was 6.3 ± 5.3 years. The mean periods of diabetes among patients with a high NLR and a normal NLR were 6.5 ± 4.9 years and 6.1 ± 5.7 years, respectively (p-value = 0.744 by unpaired t-test).

Sugar control was assessed by measuring HbA1c levels, and patients were divided into two groups based on the HbA1c cut-off value of 7% [16]. A total of 71 patients were found to have HbA1c levels of >7%, and 29 patients had HbA1c levels of <7%. The highest HbA1c level was 14.8%, and the lowest HbA1c level was 6.0%. The mean HbA1c level of the study population was 8.9 ± 2.4%. The mean HbA1c levels among patients with a high NLR and a normal NLR were 9.8 ± 2.5% and 8 ± 1.9%, respectively (p = 0.0002; unpaired t-test) (Table 2).

**Table 2:** Glycemic control and NLR HbA1c: glycosylated hemoglobin; NLR: neutrophil-to-lymphocyte ratio; N: number; SD: standard deviation; p-value: probability value

HbA1c (%)	NLR		Combined	p-value
	High	Normal		
N	48	52	100	0.0002
Mean	9.78	8.04	8.88	
SD	2.54	1.94	2.40	

A total of 58 patients had either one, two, or all of the three microvascular complications. The remaining patients did not have any microvascular complications. Nephropathy, retinopathy, and neuropathy were found in 45 patients, 28 patients, and 33 patients, respectively. A total of 21 patients had both nephropathy and retinopathy; 20 patients had both nephropathy and neuropathy; and 15 patients had both retinopathy and neuropathy. A total of eight patients had all three microvascular complications. Of the 58 patients with microvascular complications, 34 patients had a high NLR, and 24 patients had a normal NLR. Of the 42 patients without microvascular complications, only 14 had a high NLR, and 28 patients had a normal NLR. In the chi-square test, the p-value was 0.012 (Table 3).

**Table 3:** Microvascular complications and NLR NLR: neutrophil-to-lymphocyte ratio; p-value: probability value

Microvascular complications	High NLR		Normal NLR		Combined		p-value
	Number of cases	%	Number of cases	%	Number of cases	%	
Yes	34	58.62%	24	41.38%	58	100	0.012
No	14	33.33%	28	66.67%	42	100	
Total	48	48.00%	52	52.00%	100	100	

Of the 45 patients with diabetic nephropathy, there were 27 patients with a high NLR, and 18 patients with a normal NLR. Of the remaining 55 patients without diabetic nephropathy, there were 21 patients with a high NLR and 34 patients with a normal NLR (p = 0.030; chi-square test) (Table 4).

**Table 4:** Nephropathy and NLR NLR: neutrophil-to-lymphocyte ratio; p-value: probability value

Diabetics with nephropathy	High NLR		Normal NLR		Combined		p-value
	Number of cases	%	Number of cases	%	Number of cases	%	
Yes	27	60.00%	18	40.00%	45	100%	0.030
No	21	38.18%	34	61.82%	55	100%	
Total	48	48.00%	52	52.00%	100	100%	

Of the 28 patients with diabetic retinopathy, there were 19 with a high NLR, and nine with a normal NLR. Of the remaining 72 patients without diabetic retinopathy, there were 29 patients with a high NLR and 43 patients with a normal NLR (p = 0.013; chi-square test) (Table 5).

**Table 5:** Retinopathy and NLR NLR: neutrophil-to-lymphocyte ratio; p-value: probability value

Diabetics with retinopathy	High NLR		Normal NLR		Combined		p-value
	Number of cases	%	Number of cases	%	Number of cases	%	
Yes	19	67.86%	9	32.14%	28	100%	0.013
No	29	40.28%	43	59.72%	72	100%	
Total	48	48.00%	52	52.00%	100	100%	

Of the 33 patients with diabetic neuropathy, there were 21 with a high NLR, and 12 with a normal NLR. Of the remaining 67 patients without diabetic neuropathy, there were 27 patients with a high NLR and 40 patients with a normal NLR (p = 0.028; chi-square test) (Table 6).

**Table 6:** Neuropathy and NLR NLR: neutrophil-to-lymphocyte ratio; p-value: probability value

Diabetics with neuropathy	High NLR		Normal NLR		Combined		p-value
	Number of cases	%	Number of cases	%	Number of cases	%	
Yes	21	63.64%	12	36.36%	33	100%	0.028
No	27	40.30%	40	59.70%	67	100%	
Total	48	48.00%	52	52.00%	100	100%	

Of the eight patients with all three microvascular complications, seven had a high NLR, and only one had a normal NLR. A total of 92 patients did not have all three microvascular complications, and of these patients, 41 had a high NLR, and 51 patients had a normal NLR. The results were statistically significant (p = 0.020; chi-square test) (Table 7).

**Table 7:** All three microvascular complications and NLR NLR: neutrophil-to-lymphocyte ratio; p-value: probability value

Diabetics with nephropathy, retinopathy, and neuropathy	High NLR		Normal NLR		Combined		p-value
	Number of cases	%	Number of cases	%	Number of cases	%	
Yes	7	87.50%	1	12.50%	8	100%	0.020
No	41	44.57%	51	55.43%	92	100%	
Total	48	48.00%	52	52.00%	100	100%	

Receiver operating characteristic (ROC) curve analysis

**Figure 1:**
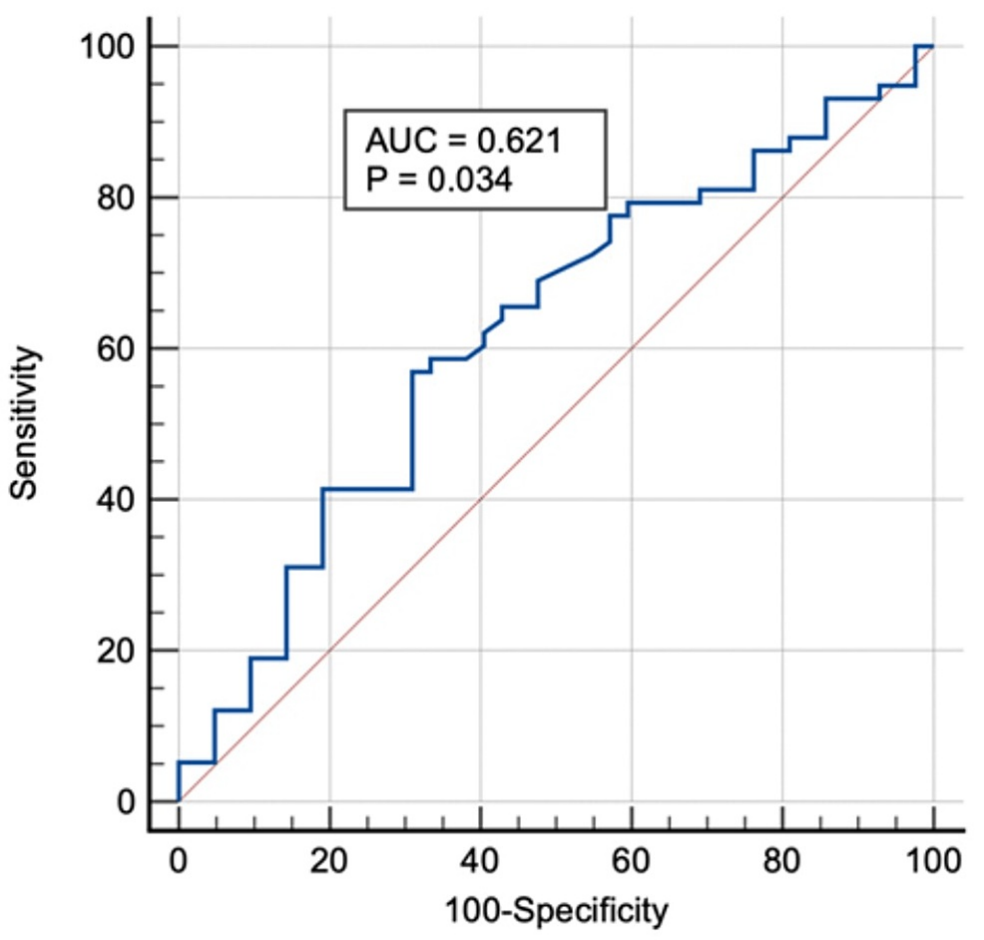
The ROC curve of NLR as a predictor of total microvascular complications ROC: receiver operating characteristic: AUC: area under the ROC curve; NLR: neutrophil-to-lymphocyte ratio

The area under the ROC curve (AUROC) of the NLR for predicting total microvascular complications was 0.621 (95% confidence interval (CI): 0.519-0.716; p = 0.034). An NLR with a cut-off value of four demonstrated 56.9% sensitivity and 69.05% specificity in predicting total microvascular complications (Figure 1).

The AUROC of the NLR for predicting diabetic nephropathy was 0.637 (95% CI: 0.535-0.731; p = 0.015). An NLR with a cut-off value of 4.79 demonstrated 44.44% sensitivity and 80.00% specificity in predicting diabetic nephropathy. The AUROC of the NLR for predicting diabetic retinopathy was 0.651 (95% CI: 0.549-0.744; p = 0.014). An NLR with a cut-off value of four demonstrated 67.86% sensitivity and 62.50% specificity in predicting diabetic retinopathy. The AUROC of the NLR for predicting diabetic neuropathy was 0.585 (95% CI: 0.482-0.683; p = 0.149). An NLR with a cut-off value of 2.83 demonstrated 78.79% sensitivity and 49.35% specificity in predicting diabetic neuropathy.

Logistic regression analysis

Multivariate logistic regression was performed to analyze the association between the NLR and microvascular complications. There was a significant association between the NLR and overall microvascular complications (OR: 2.83, 95% CI: 1.23-6.48) (Table 2). In addition, the NLR was significantly correlated with diabetic nephropathy (OR: 2.55, 95% CI: 1.131-5.748), diabetic retinopathy (OR: 3.13, 95% CI: 1.244-7.873), and diabetic neuropathy (OR: 2.59, 95% CI: 1.095-6.133) (Table 8).

**Table 8:** Logistic regression analysis of NLR and microvascular complications, diabetic nephropathy, diabetic retinopathy, and diabetic neuropathy NLR: neutrophil-to-lymphocyte ratio; p-value: probability value; SD: standard error; OR: odds ratio; CI: confidence interval

	Regression coefficient (B)	SE	p-value	OR	95% CI for OR	
					Lower	Upper
NLR and microvascular complications	1.041	0.422	0.013	2.833	1.238	6.481
NLR and diabetic nephropathy	0.936	0.414	0.024	2.550	1.131	5.748
NLR and diabetic retinopathy	1.141	0.470	0.015	3.130	1.244	7.873
NLR and diabetic neuropathy	0.952	0.439	0.030	2.592	1.095	6.133

## Discussion

The objective of the study was to determine the NLR of diabetic patients and examine its association with microvascular complications. The measurement of NLR is a simple and low-cost method that can be performed even in resource-limited areas to predict microvascular complications in the early stages so that measures can be taken to prevent further deterioration.

We assessed the NLR of diabetics and its association with gender, age, duration of diabetes, blood sugar control, and microvascular complexities. The mean age of the study population was 56.28 ± 13.24 years, and the age group of 41-50 years had the highest number of subjects. No association was observed between the age of patients and a high NLR (p = 0.126). This finding is in agreement with that of Chittawar et al., who also reported no relationship between the NLR and the age of patients [17]. There were 64 males and 36 females in our study, and there was no association between gender and a high NLR. The mean period of diabetes in our study was 6.32 ± 5.32 years. There was no significant association between the duration of diabetes and the NLR (p = 0.744). However, in a previous study by Chittawar et al., there was a significant association between the NLR and the duration of diabetes [17].

A statistically significant association was found between poor glycemic control (HbA1c >7%) and a high NLR (p = 0.0002). The results were in agreement with the findings of Sefil et al., who also reported a positive association between the NLR and HbA1c levels [18]. Similarly, a study conducted by Moursy et al. found that the NLR was positively correlated with HbA1c levels [8].

In our study, 58 patients had one or more microvascular complications, and among them, 34 patients were found to have a high NLR. The association between the presence of microvascular complications and a high NLR was statistically significant (p = 0.012). A study conducted by Ozturk et al. also found that patients with microvascular complications had a higher NLR than that of patients without complications and controls [19]. Similarly, a study by Moursy et al. reported similar results [8].

We also assessed the microvascular complications of patients separately in three groups. Nephropathy, retinopathy, and neuropathy were present in 45 patients, 28 patients, and 33 patients, respectively. Of the 45 patients with nephropathy, 27 patients had a high NLR. The association between the presence of diabetic nephropathy and a high NLR was statistically significant (p = 0.030). Our results are consistent with those of a study in India conducted by Khandare et al., who examined 115 patients with type 2 diabetes mellitus and found a correlation between a high NLR and diabetic nephropathy [20]. Another study by Singh et al. reported similar results, showing that the NLR could be considered a reliable predictive marker in diabetic nephropathy [21]. In a study by Khan et al., the NLR of subjects with type 2 diabetes and increased albuminuria was markedly elevated [22].

Of the 28 patients with retinopathy, 19 had a high NLR. The association between the presence of diabetic retinopathy and a high NLR was statistically significant (p = 0.013). In agreement with our findings, Ulu et al. identified the NLR as a predictive marker for subjects with diabetic retinopathy [23]. Yue et al. also found that the NLR was considerably higher among patients with diabetic retinopathy compared with diabetics without retinopathy [24]. In contrast, a study conducted by Ciray et al. reported no association between a high NLR and diabetic retinopathy [25]. However, two recent studies found a positive association between NLR and diabetic retinopathy. In 2020, Li et al. identified an elevated NLR as a dependable marker for diabetic retinopathy [26]. In a multivariate analysis conducted by He et al. in 2022, the NLR was related to the risk of diabetic retinopathy [27].

Of the 33 patients with neuropathy in our study, 21 had a high NLR. The association between the presence of diabetic neuropathy and a high NLR was statistically significant (p = 0.028). This finding is in agreement with that of Liu et al., who showed a positive correlation between a high NLR and diabetic peripheral neuropathy [28]. In the same year, Xu et al. reported a significant association of the NLR with diabetic peripheral neuropathy [29]. Another recent study by Chen et al. concluded that the NLR could be used as a prognosticator for diabetic peripheral neuropathy in type 2 diabetes mellitus [30]. However, a study conducted in India by Chittawar et al. showed different results, where the NLR was not a reliable prognosticator for the occurrence of diabetic neuropathy [17].

Our study also showed that 21 patients had both nephropathy and retinopathy, of whom 15 had a high NLR. The association between the presence of both diabetic neuropathy and retinopathy and a high NLR was statistically significant (p = 0.016). A total of 20 patients had both nephropathy and neuropathy, of whom 14 patients (70%) had a high NLR. The association between the presence of both diabetic neuropathy and retinopathy and a high NLR was statistically significant (p = 0.028). A total of 15 patients had both retinopathy and neuropathy, of whom 11 had a high NLR. The association between the presence of both diabetic neuropathy and retinopathy and a high NLR was statistically significant (p = 0.034).

In our study, eight patients had all three microvascular complications, of whom seven had a high NLR. The association between the presence of diabetic nephropathy, neuropathy, and retinopathy and a high NLR was statistically significant (p = 0.020). The result is consistent with that of a study conducted by Moursy et al., who demonstrated the high NLR of patients with diabetes and more than one microvascular complication compared with those with only one microvascular complication [8].

A limitation of our study is that logistic regression could not be adjusted for major risk factors such as hypertension, dyslipidemia, obesity, and smoking (a cardiovascular risk factor), as we do not have sufficient data. A strength of the study is that it demonstrated that the NLR could predict total microvascular complications with a sensitivity of 56.9% and a specificity of 69.5% (OR: 2.83, 95% CI: 1.23-6.48).

## Conclusions

Overall, the findings suggest that a high NLR may be observed in cases of diabetes mellitus involving one or more microvascular complications and poor glycemic control. The NLR can be used as a rapid marker for diabetics to predict overall microvascular complications. A high NLR indicates higher odds of having several microvascular complications (diabetic nephropathy, diabetic retinopathy, and diabetic neuropathy). The findings of our study are consistent with those of several recently published studies showing the NLR as a marker of microvascular complications in diabetes.
